# Male Weaponry in a Fighting Cricket

**DOI:** 10.1371/journal.pone.0003980

**Published:** 2008-12-24

**Authors:** Kevin A. Judge, Vanessa L. Bonanno

**Affiliations:** 1 Department of Biology, University of Toronto Mississauga, Mississauga, Ontario, Canada; 2 Department of Biology, Laurentian University, Sudbury, Ontario, Canada; University of Bristol, United Kingdom

## Abstract

Sexually selected male weaponry is widespread in nature. Despite being model systems for the study of male aggression in Western science and for cricket fights in Chinese culture, field crickets (Orthoptera, Gryllidae, Gryllinae) are not known to possess sexually dimorphic weaponry. In a wild population of the fall field cricket, *Gryllus pennsylvanicus*, we report sexual dimorphism in head size as well as the size of mouthparts, both of which are used when aggressive contests between males escalate to physical combat. Male *G. pennsylvanicus* have larger heads, maxillae and mandibles than females when controlling for pronotum length. We conducted two experiments to test the hypothesis that relatively larger weaponry conveys an advantage to males in aggressive contests. Pairs of males were selected for differences in head size and consequently were different in the size of maxillae and mandibles. In the first experiment, males were closely matched for body size (pronotum length), and in the second, they were matched for body mass. Males with proportionately larger weaponry won more fights and increasing differences in weaponry size between males increased the fighting success of the male with the larger weaponry. This was particularly true when contests escalated to grappling, the most intense level of aggression. However, neither contest duration nor intensity was related to weaponry size as predicted by models of contest settlement. These results are the first evidence that the size of the head capsule and mouthparts are under positive selection via male-male competition in field crickets, and validate 800-year-old Chinese traditional knowledge.

## Introduction

Darwin [1] proposed that male weaponry could evolve as a result of aggressive physical competition among males over reproductive access to females. The scientific literature abounds with examples of male traits that are used in direct combat between males for mates, including: antlers and horns in ungulates ([2], [3], reviewed in [4]), canines in primates [5], avian spurs ([6], reviewed in [4]), heads in lizards [7], chelae in crabs [8], horns and mandibles in beetles ([9], [10], [11], [12], reviewed in [4]), mandibles and maxillae in tree weta [13], [14], [15], [16], forceps in earwigs ([17], [18], but see [19], [20]), antlers, eyestalks [21] and forelegs in flies [22], [23], chelate pedipalps in pseudoscorpions [24], and forelegs in thrips [25]. In each of these groups, weaponry is either limited to, or larger in males than females, and after correcting for body size differences between combatants, the male with the relatively larger weapon(s) usually wins in aggressive combat with smaller males (see refs. above). Therefore, weapon size is an index of a male's resource holding potential (RHP [26]) since it influences the outcome of aggressive interactions.

Several authors have argued that assessment strategies should evolve that would allow individuals to terminate a contest before incurring the costs of losing in direct physical combat with a stronger opponent [27], [28], [26]. Models of contest settlement mostly fall into two general categories, those in which contestants' decisions to persist are dependent on: 1) relative RHP [29], [30], [31], [32], or 2) their own RHP [33], [34]. A third type, the cumulative assessment model [35], posits that combatants persist until some threshold of the loser (e.g. energy expenditure, damage) is reached at a rate determined by the RHP of both contestants. All of these models predict that weaponry, as an index of RHP, will be correlated with contest duration.

Field crickets (Orthoptera, Gryllidae, Gryllinae) are model organisms for the study of male-male aggression, including models of RHP assessment (e.g. [36], [37], [38], [39], [40], [41], [42], [43], [44]). However, aside from the sexual dimorphism in specialized sound producing structures on the forewings, used by males in part for an aggressive function [36], few studies have reported any sexually dimorphic morphological trait that might be classed as weaponry (but see below).

In nature, male field crickets defend burrows from which they call to attract females [45]. Males are extremely aggressive towards each other [1], [36], and vigorously defend their territories from intruding males [45]. Contests between males proceed through a highly stereotyped series of aggressive behaviours, with the most escalated contests ending with males head-butting each other and grappling with their mouthparts [36]. Female field crickets can also be aggressive, however they rarely grapple with their mouthparts [46]. Thus, the head and mouthparts (both maxillae and mandibles) of male field crickets have likely been the targets of strong sexual selection mediated through aggressive physical combat.

Several lines of evidence suggest that the heads, maxillae and mandibles of male field crickets are sexually selected through male-male competition. First, Walker et al. [47] showed that for *Acheta domesticus*, a commercially-raised species, males had larger heads (and presumably also larger maxillae and mandibles) than females. Observations by Alexander [48] had much earlier hinted at this pattern in two other North American species, *Gryllus pennsylvanicus* and *G. firmus* (see [49], [50] for similar observations on African gryllines). This pattern of sexual dimorphism is likely widespread in grylline crickets (D. Otte, pers. comm.). Second, in other ensiferan Orthoptera (Anostostomatidae, *Hemideina* spp.), the size of enlarged mandibles [14], [15], [51] is positively correlated with male fighting success ([16], but see [44]). Third, gambling on the outcome of fights between male grylline crickets has occurred in China since the Sung Dynasty (A.D. 960–1278) [52], [53], [54], [55], [56], [57], [58]. This traditional practice has resulted in a list of traits thought to be possessed by superior fighters [52], [53], [54]. An early work on cricket fighting by Chia Szu-Tao in the thirteenth century states that the best fighters have large heads ([52]; transl. I. S. Chan). More recently, Berthold Laufer, the noted American sinologist [59], reported, “The good fighters, according to Chinese experts, are recognized by their loud chirping, their *big heads and necks*…” (p. 18, [53], emphasis added; see also [54]). Even today, male field crickets with big heads are valued as good fighters [60], [61].

In this paper we test the hypothesis that the size of male heads, maxillae and/or mandibles (i.e. weaponry) of the fall field cricket, *G. pennsylvanicus*, has been shaped by sexual selection through male-male aggression. We first quantify the pattern of sexual dimorphism in head size (as well as maxillae and mandible size) alluded to by Alexander [48], and we test whether variation in any of these morphological dimensions is related to success in staged agonistic contests between size-matched males. We predict that, after experimentally controlling for male body size differences: 1) males with larger weaponry will win more staged fights than males with smaller weaponry, and 2) an increase in the difference in weaponry size between males will increase the likelihood of a win for the male with the larger weaponry. Briffa [44] found that mandible asymmetry, rather than length, was related to fighting success, so we tested this hypothesis as well. Although our experiment was not designed to distinguish among different theoretical models of agonistic contest settlement (see above), we also tested a common prediction of these models that the size of participants' weaponry affects the duration of contests.

## Methods

### Study Species

*G. pennsylvanicus* is a univoltine, egg-diapausing field cricket widespread across much of eastern North America [62], [63]. To analyse sexual dimorphism, we collected individuals from the grounds of the University of Toronto Mississauga (43°32′50.51″N, 79°39′37.80″W) from 13 August to 21 September 2003. All animals used in the aggressive contests were third generation offspring of adult *G. pennsylvanicus* caught from the same location during August and September of 2002.

### Animal Rearing

Juvenile crickets were kept in large plastic containers (48 cm long, 35 cm wide, 31 cm high) at 25°C, 70% relative humidity and a light cycle of 12 hr light: 12 hr dark. All were fed Purina® Cat Chow (ground pellets for the first two to three weeks of life and whole pellets later on) and provided with water in cotton-plugged vials. We added new food every three to four days and changed water vials as needed. Layers of egg cartons provided shelter. To reduce cannibalism of smaller individuals, we moved larger nymphs to a separate bin. We isolated penultimate-instar nymphs in individual containers (9 cm diameter, 8 cm high) with two pieces of food, a cotton-plugged water vial and a small piece of egg carton. Food in individual containers was changed weekly and water was changed at least bi-weekly or more frequently if needed. Every day we checked individually housed nymphs for newly molted adults. This allowed us to assign ages to all individuals.

### Sexual Dimorphism in Morphology

**Figure 1:**
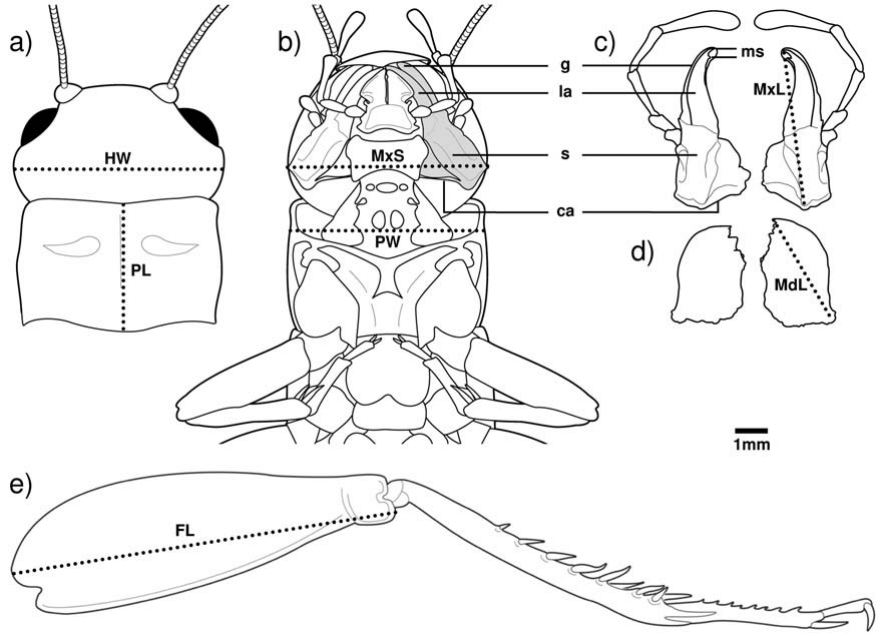
A male cricket showing the: a) dorsal view of the head capsule and pronotum, b) ventral view of the head capsule and pronotum (head has been tilted dorsally to expose ventral mouthparts), c) ventral view of the right and left maxillae, d) ventral view of the right and left mandibles, and e) left hind leg. All drawings are at the same scale. Morphological measurements are shown as dotted lines and abbreviations are as follows: HW = head width, PL = pronotum length, MxS = maxillae span, PW = pronotum width, MxL = maxilla length, MdL = mandible length, and FL = femur length. The left maxilla, excluding the palp, is shaded grey in b) and labeled with abbreviations indicating the various parts in both b) and c): la = lacinia, g = galea, s = stipes, ca = cardo, and ms = maxadentes (after [81]). Note that the second maxadentes on the right maxilla shows an abnormality in being slightly shorter and blunted. Drawings by Janice J. Ting.

We collected 151 males and 75 females in 2003 and euthanized them by freezing. These individuals were stored frozen until we measured: left and right femur length, pronotum length, pronotum width (spanning the ventral margins across the neck membrane and cervical sclerites), head width, maxillae span (the transverse distance between the dorsal edge of the cardo-stipes articulation of the right and left maxillae viewed ventrally), left and right maxilla length (from the ventral edge of the cardo-stipes articulation to the distal tip of the lacinia), and left and right mandible length (from the lateral articulation to the distal tip) (Fig. 1). We calculated mandible length asymmetry as left minus right following Briffa [44]. Measurements were taken using NIH Image 1.62 on images captured from a camera mounted on a dissecting microscope. The focal height of the microscope was fixed for each measurement, which ensured a high degree of repeatability (>99%) for each (K. A. Judge, unpubl. data).

In addition to the linear dimensions of male and female morphology, we were also able to collect observations of deformed, damaged or missing body parts from which the above measurements were taken.

### Experiment 1: Aggressive Contests Controlling for Pronotum Length

One to two days following the adult molt, we measured each male's head width and pronotum length. We did this by first restraining them on the surface of a petri dish under a small piece of plastic wrap weighed down by a plastic ring. This allowed us to position the cricket so that the frontal plane was perpendicular to the line of sight under a dissecting microscope. As above, all measurements were taken using NIH Image 1.62. To test whether relative head/mouthpart size has an effect on the outcome of male-male aggressive contests, we formed pairs of males matched for age and body size (pronotum length), but differing in head width, and staged a series of male-male contests. Because head width is strongly and positively correlated with maxillae span, mean maxilla length and mean mandible length (see Results), male pairs that differed in head width also differed in the size of their mouthparts. Each male took part in only one aggressive contest.

Within each pair of males, individuals were randomly assigned one of two identification colours, and a small dot of nail polish of the appropriate colour was applied to the pronotum 24 hours before the aggressive contest (to allow time to recover from handling). All males were weighed to the nearest 0.1 milligram using a Mettler AE 50 balance approximately one hour before the contests were conducted.

The contest arena was a square glass box with an open top (12.5 cm long, 12.5 cm wide, 18.0 cm high). Coarse sand covered the bottom, and brown paper covered the outside surface to minimize visual disturbances. A removable opaque plastic wall divided the box into two equal triangles. Before each trial, the interior walls and divider were rubbed with an ethanol-soaked cotton ball, and the sand base was shaken and tossed to minimize and disperse any pheromonal cues left by the previous contest. We recorded all trials from directly above the arena with a SONY® digital video camera (model # DCR-TRV740). For each contest, we introduced each male of a pair into opposite sides of the box, and after a two-minute acclimatization period, we started the video recording and withdrew the divider. Recording was halted after 10 min and the males were returned to their individual containers. Males were weighed after the contest, euthanized by freezing and then placed in 95% ethanol for subsequent measurement of mandible length, maxilla length and maxillae span.

### Experiment 2: Aggressive Contests Controlling for Body Mass

As body mass differences are known to affect the outcome of aggressive contests in field crickets [39] and were not experimentally controlled in experiment 1, we conducted a second experiment in which males were matched for body mass instead of pronotum length. All males were handled and marked as in experiment 1, except that we weighed males several hours before the aggressive contests and then matched pairs of males for similar body mass but different head width.

### Video Analysis

We transferred the video of each contest to DVD on a Macintosh G5 using the software iMovie® (Apple Computer, Inc.). For each trial we recorded the duration of the aggressive contest – from first contact until the contest ended (one male retreated from its opponent following a break in the aggressive contest) – and the contest victor, determined as the individual that tremulated or stridulated, and chased its opponent. In most cases, the identity of contest winners is determined with little difficulty (e.g. [42]). We also noted the maximum level of aggression (contest intensity) attained in each contest. For this we used a modified version of the categorical scale of aggression level used by Hofmann and Schildberger [41]. The categories used are as follows: 0 = mutual avoidance, 1 = immediate dominance, 2 = mutual antennation, 3 = unilateral maxillae/mandible spreading, 4 = bilateral maxillae/mandible spreading, and 5 = grappling. The only difference between this scale and the one used by Hofmann and Schildberger [41] is the collapse of their last two categories (5 = mandible pushing and 6 = grappling) into one, because of the difficulty in distinguishing these in the video.

Although we did not explicitly design the video analysis to be truly blind to differences in head width, in practice, the experimenter watching the video was unaware of this information.

### Statistical Analysis

We tested whether male and female *G. pennsylvanicus* could be distinguished on the basis of morphology by conducting a discriminant function analysis using head width, maxillae span, mean maxilla length, mean mandible length, mandible length asymmetry, pronotum width, pronotum length and mean femur length as predictors of membership in either sex. Also, given that male field crickets are more likely than females to escalate aggressive encounters [46], males may suffer more damage to their exoskeleton than females. Therefore, sex differences in the proportion of wild-caught individuals with deformed, damaged or missing body parts (head capsule, maxillae, mandibles, pronotum, hind legs) were tested using a normal approximation to the chi-square test [64].

Since all of the head and mouthpart size measurements are positively correlated with each other in males (see Results), we also conducted a principal components analysis (PCA) on the five weaponry dimensions to reduce them to a set of orthogonal principal components that captured variation in male weaponry. This allowed us to avoid committing a Type I error through repeated statistical hypothesis testing of each of the individual weaponry dimensions.

We used binomial tests [64] to evaluate the prediction that males with larger weaponry would win contests more often than males with smaller weaponry. To test for an effect of the magnitude of the difference in weaponry size on contest outcome, we used stepwise logistic regression. We first randomly selected one focal male from each pair and then scored the outcome of each contest as to whether the focal male won (1) or the focal male lost (0). We then calculated the difference between focal and rival male in each significant principal component dimension that resulted from the PCA of the weaponry dimensions (head width, maxillae span, mean maxilla length, mean mandible length and mandible asymmetry). Differences between males in each of the weaponry dimensions thus range from negative values (focal male smaller than rival) to positive values (i.e. focal male larger than rival), and were entered into both forward and backward stepwise logistic regressions as predictors of contest outcome. Because of individual variation in body shape, controlling one dimension of size in each experiment necessarily left others imperfectly controlled. For this reason we also included the difference between males in an uncontrolled size dimension (i.e. body mass in experiment 1 and pronotum length in experiment 2) as a predictor in the above stepwise logistic regressions to test for the effect of uncontrolled variation in body size on contest outcome.

Models of contest settlement make specific predictions regarding the relationship between male RHP traits and contest duration and intensity (reviewed in [65]). Therefore we tested for an effect of weaponry size on both contest duration and intensity by conducting bivariate correlations between each of these two variables and values of weaponry size for winning males, losing males, larger males and smaller males (following [65]).

Statistical tests were carried out at alpha = 0.05 using SPSS 10 (SPSS Inc.). Although the vast majority of studies have shown that males with larger weaponry are better fighters (see Introduction), to be conservative we used two-tailed tests throughout the manuscript.

## Results

### Sexual Dimorphism in Morphology

**Figure 2:**
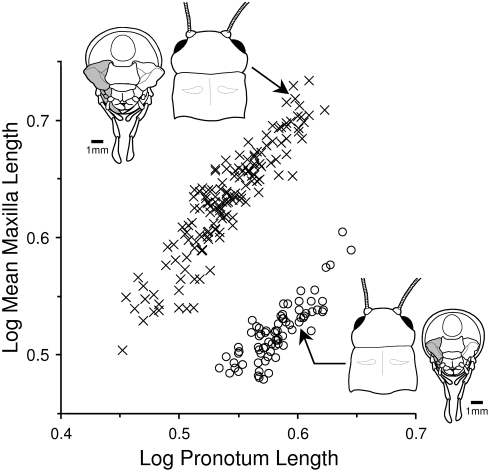
Scatterplot of log mean maxilla length versus log pronotum length showing the sexual dimorphism in mean maxilla length in a sample of 151 males (X) and 75 females (O). Drawings are of a representative male (upper left) and female (lower right) showing the dorsal view as in Fig. 1a as well as the posterior view of the ventral surface of the head (left maxilla is shaded grey as in Fig. 1b). Arrows point to the individuals whose measurements are depicted. Drawings by Janice J. Ting.

All morphological dimensions (see Fig. 1) were highly positively correlated with each other except for correlations involving mandible length asymmetry, which were weaker and not statistically significant in females (Table S1). Males were larger than females in all morphological dimensions that involved the head and mouthparts (Table 1), whereas females had longer pronota and femora (Table 1). Discriminant function analysis resulted in a linear combination of the eight morphological traits (i.e. the discriminant function) that accurately distinguished wild-caught male and female *G. pennsylvanicus* 100% of the time (Wilks' Lambda = 0.052, χ ^2^ = 651.848, df = 8, p<0.001). Mean maxilla length and pronotum length were the two morphological traits that distinguished males and females most strongly (i.e. loaded most heavily on the discriminant function, but with opposite signs; Table 1): males had longer maxillae (mean±SE = 4.30±0.04 mm) but shorter pronota (3.48±0.02 mm) than females (mean maxilla length: 3.31±0.02 mm; pronotum length: 3.84±0.02 mm) (Table 1, Fig. 2).

**Figure 3:**
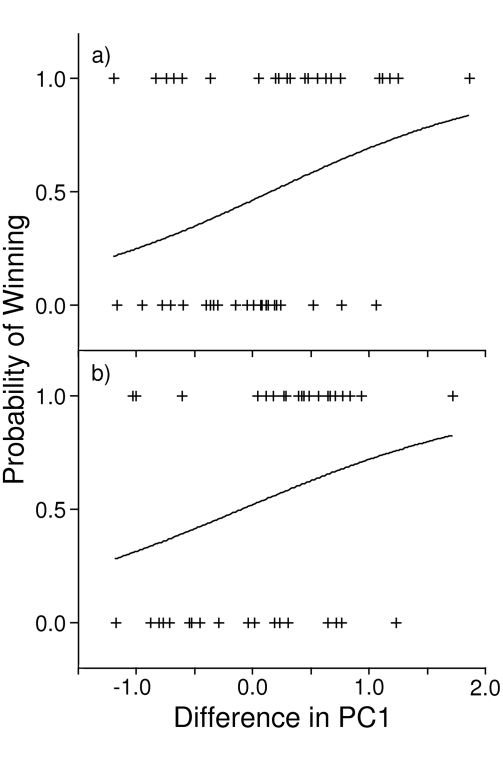
Probability that the focal male would win a fight versus the difference in PC1 between the focal male and his rival for: a) experiment 1, and b) experiment 2. Positive values of PC1 difference mean that the focal male had larger weaponry than his rival, whereas negative values indicate that the focal male had smaller weaponry than his rival. Observed values are represented by pluses (+) and the solid lines are plots of the logistic models.

**Table 1:** Summary of sexual differences in eight homologous morphological measurements in wild-caught *G. pennsylvanicus*.

Measurement	Male N	Male Mean±SE (mm)	Male Range (mm)	Male CV (%)	Female N	Female Mean±SE (mm)	Female Range (mm)	Female CV (%)	t†	Loading‡
Maxillae Span	151	5.68±0.05	4.16–7.03	9.82	75	4.68±0.03	4.07–5.46	5.83	18.044***	0.228
Mean Maxilla Length	151	4.30±0.04	3.19–5.42	10.53	75	3.31±0.02	3.01–4.02	5.86	23.019***	0.283
Mean Mandible Length	151	3.06±0.03	2.21–3.90	10.80	75	2.42±0.02	2.19–2.93	5.99	20.176***	0.249
Mandible Length Asymmetry L–R	151	0.12±0.003	−0.01–0.21	30.46	75	0.07±0.003	0.01–0.12	44.12	11.596***	0.181
Head Width	151	5.71±0.04	4.43–6.95	8.75	75	5.38±0.03	4.91–6.21	5.07	6.403***	0.083
Pronotum Width	151	5.41±0.04	4.25–6.42	8.30	75	5.30±0.04	4.65–6.17	5.76	2.069*	0.028
Pronotum Length	151	3.48±0.02	2.83–4.20	8.22	75	3.84±0.02	3.42–4.42	5.52	−10.570***	−0.149
Mean Femur Length	151	10.45±0.06	8.47–12.83	6.96	75	10.97±0.07	10.02–13.13	5.52	−5.692***	−0.084

† All differences (except for mandible length asymmetry and mean femur length) were tested using Student's t-tests for unequal variances after Levene's tests for equality of variances detected significant heteroscedasticity.

‡ Loadings give the correlations between each measurement and the discriminant function separating males and females.

* p<0.05.

*** p<0.001.

Males were significantly more likely to have the tips of their maxillae (lacinia) blunted or apparently snapped off at the maxadentes (e.g. see Fig. 1), than females (14/151 males, 0/76 females, Z = 2.430, p = 0.015), whereas there was no significant difference in the proportions of males and females that were missing one hind femur (4/151 males, 6/75 females, Z = 1.498, p = 0.134). We did not detect any abnormalities in head capsules or mandibles, and only one male's pronotum was misshapen.

### Experiment 1: Aggressive Contests Controlling for Pronotum Length

By establishing pairs of males with similar pronotum lengths but different head widths, we were also able to produce pairs that varied in maxillae span, mean maxilla length, mean mandible length and mandible length asymmetry (Table 2).

**Table 2:** Summary of the mean absolute differences in morphology between males within pairs in both experiment 1 (N = 52 pairs) and experiment 2 (N = 42 pairs).

Measurement	Experiment 1 Mean±SE	Experiment 1 Range	Experiment 2 Mean±SE	Experiment 2 Range
Maxillae Span (mm)	0.27±0.02	0.03–0.79	0.32±0.03	0.01–0.64
Mean Maxilla Length (mm)	0.19±0.02	0.01–0.58	0.23±0.02	0.03–0.68
Mean Mandible Length (mm)	0.13±0.01	0.00–0.43	0.17±0.02	0.01–0.88
Mandible Length Asymmetry L-R (mm)	0.03±0.003	0.00–0.10	0.03±0.004	0.00–0.12
Head Width (mm)	0.21±0.02	0.00–0.52	0.23±0.02	0.01–0.67
Pronotum Length (mm)	0.04±0.01	0.00–0.21	0.18±0.02	0.00–0.67
Body Mass (mg)	48±5	0–146	10±1	0–42

Of 52 completed aggressive contests, we could unambiguously determine a winner within the ten-minute time limit in 47. In five contests without a clear winner, there was no overt aggression and the males either courted each other continuously or failed to make contact.

A PCA of head width, maxillae span, mean maxilla length, mean mandible length and mandible length asymmetry (i.e. weaponry dimensions) resulted in two principal components (PCs) that explained over 97% of the variance in the five weaponry dimensions (Table 3). All weaponry dimensions except for mandible length asymmetry loaded strongly and positively on PC1, and weakly and negatively on PC2, whereas mandible length asymmetry loaded strongly and positively on PC2 (Table 3). We therefore interpret PC1 as a measure of overall weaponry size and PC2 as mandible length asymmetry.

**Table 3:** Summary of the principal components analysis of the five measurements of head and mouthpart dimensions for experiment 1 (N = 104 males) and experiment 2 (N = 84 males); values are the loading factors for each of the measurements on each of the two principal components, followed by the percent of the total variance explained by each PC.

Measurement	Experiment 1 PC1	Experiment 1 PC2	Experiment 2 PC1	Experiment 2 PC2
Maxillae Span	0.980	−0.129	0.980	−0.122
Mean Maxilla Length	0.987	−0.079	0.983	−0.116
Mean Mandible Length	0.986	−0.054	0.979	−0.041
Mandible Length Asymmetry	0.435	0.900	0.428	0.904
Head Width	0.961	−0.138	0.973	−0.116
Variance Explained	80.4%	17.1%	80.3%	17.2%

Although the overall proportion of males that won aggressive contests and were larger than their rival on PC1 was not significantly different from random chance (28/47 = 60%, binomial p = 0.243), the magnitude of the difference in PC1 between males did significantly affect contest outcome (χ^2^ = 4.271, p = 0.039, Nagelkerke R^2^ = 0.116). As the difference in PC1 increased (i.e. weaponry of focal male became bigger), so too did the likelihood that the focal male would win the aggressive contest (slope±SE = 0.961±0.495, odds ratio = 2.6) (Fig. 3a). Only 24 of the 47 males (51.1%) who won aggressive contests scored higher than their rival on PC2 (binomial p = 1.000), and the magnitude of the difference in PC2 between males had no effect on contest outcome (χ^2^ = 0.365, p = 0.546, Nagelkerke R^2^ = 0.010). Although we could not experimentally control for asymmetry in body mass (Table 2), including the difference in body mass in stepwise logistic regressions with each of the above predictors did not alter the final logistic models reached.

Contest duration was not normally distributed (Kolmogorov-Smirnov test statistic = 0.185, p<0.001), and although a square-root transformation restored normality, we proceeded using nonparametric Spearman rank correlations for ease of explanation since parametric correlations using transformed data did not change our interpretation (data not shown). Neither PC1 or PC2 scores for winning males, losing males, larger males or smaller males were related to contest duration (all p>0.650) or contest intensity (all p>0.117), although contest duration was positively correlated with contest intensity (Spearman's rho = 0.473, p<0.001).

### Experiment 2: Aggressive Contests Controlling for Body Mass

We established pairs of males that were similar in body mass and varied in head width difference as well as differences in mouthpart dimensions (Table 2). There was a clear fight winner in 39 of 42 contests; three contests with continuous mutual male courtship were excluded.

As in experiment 1, PCA resulted in two PCs that explained over 97% of the variance in the five weaponry dimensions; PC1 is representative of overall weaponry size, and PC2 of mandible length asymmetry (Table 3).

In 27 of 39 (69%) aggressive contests with a clear winner, the male that scored higher on PC1 won significantly more aggressive contests than expected by chance (binomial p = 0.024). And although the magnitude of the difference between males in PC1 did not significantly affected the likelihood that the larger male would win (χ^2^ = 3.175, p = 0.075, Nagelkerke R^2^ = 0.104), the trend in the effect was similar to experiment 1: as the difference in PC1 increased, the likelihood that the focal male would win the aggressive contest also increased (slope±SE = 0.859±0.504, odds ratio = 2.4) (Fig. 3b). Males that scored higher on PC2 (i.e. had more asymmetric mandibles) than their rivals won fewer fights than would be expected by chance (13/39 = 33%, binomial p = 0.053), although the magnitude of the difference in PC2 between males had no significant effect on the outcome of aggressive contests (χ^2^ = 2.138, p = 0.144, Nagelkerke R^2^ = 0.071). Including the difference in pronotum length between males (see Table 2) as a predictor in backwards and forwards stepwise logistic regressions with each of the above predictors did not change the final logistic models.

Contest duration was again not normally distributed (K-S test statistic = 0.141, p = 0.048), and we proceeded with nonparametric correlations as in experiment 1. Only the PC2 score of the losing male was significantly correlated with contest duration (Spearman's rho = −0.355, p = 0.026; all other p>0.172). None of the correlations with contest intensity were statistically significant, although there was a trend for PC1 scores of winning males to be positively correlated with contest intensity (Spearman's rho = 0.300, p = 0.064; all other p>0.148). Contest duration was not significantly correlated with contest intensity (Spearman's rho = 0.110, p = 0.505).

### Experiments 1 and 2 Pooled

To further investigate the roles of male head and mouthpart size as weapons and/or signals of RHP, we pooled data from our two experiments after finding no significant Experiment by Weaponry interaction effects on any of the following response variables: contest outcome, contest duration or contest intensity (Table S2).

Difference in PC1 continued to predict the outcome of contests, with the larger male both: a) winning significantly more contests (55/86 = 64%, binomial p = 0.013), and b) being increasingly likely to win with greater disparity in weaponry size (χ^2^ = 7.431, p = 0.006, Nagelkerke R^2^ = 0.110, slope±SE = 0.910±0.352, odds ratio = 2.5). Difference in PC2 was not related to contest outcomes (more asymmetric male won: 37/86 = 43%, binomial p = 0.235).

**Figure 4:**
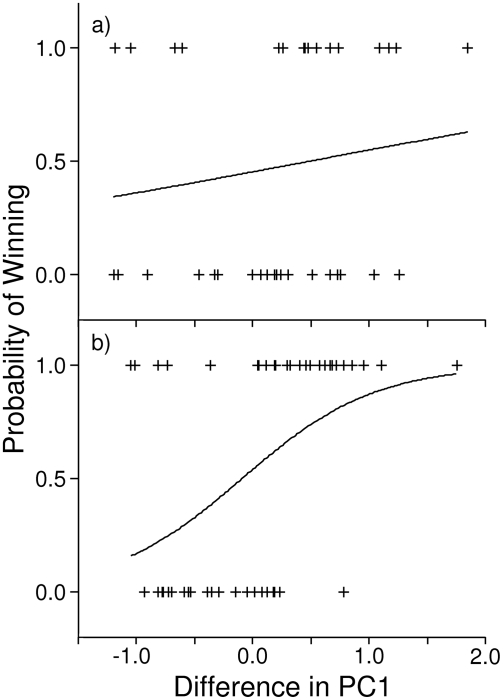
Probability that the focal male would win a fight versus the difference in PC1 between the focal male and his rival for contests that: a) did not escalate to grappling, and b) escalated to grappling. Positive values of PC1 difference mean that the focal male had larger weaponry than his rival, whereas negative values indicate that the focal male had smaller weaponry than his rival. Observed values are represented by pluses (+) and the solid lines are plots of the logistic models.

Agonistic encounters between male crickets can be divided into two broad intensity categories: 1) those that did not escalate to grappling (aggression levels 1 to 4, Table S3, N = 38) where males did not have the opportunity to use their heads and mouthparts as weapons, but where these may still have fulfilled a signaling function, and 2) those that escalated to physical combat (aggression level 5, Table S3, N = 48) where male heads and mouthparts may have performed as signals, weapons or both. We found that neither PC1 nor PC2 predicted the outcome of contests that did not escalate to grappling (larger male won [PC1]: 19/38 = 50%, binomial p = 1.000; more asymmetric male won [PC2]: 17/38 = 45%, binomial p = 0.627). However, in contests where males grappled, males with larger scores on PC1 won significantly more fights (36/48 = 75%, binomial p = 0.001) and as the magnitude of the difference in PC1 increased, so too did the probability that the larger male would win (χ^2^ = 10.731, p = 0.001, Nagelkerke R^2^ = 0.269, slope±SE = 1.764±0.614, odds ratio = 5.8; Fig. 4b). As before, differences in PC2 were not related to contest outcomes (more asymmetric male won: 20/48 = 42%, binomial p = 0.312).

Contest duration was not significantly correlated with the PC1 and PC2 scores of the winning male, losing male, larger male or smaller male (Table S4). Neither were any of these correlations statistically significant when we considered only the duration of the pre-grapple or the grapple phase of each contest (data not shown). Although contest intensity was positively correlated with winning male PC1 and smaller male PC1, these correlations were not statistically significant after correcting for multiple comparisons (Table S4). Contest duration was positively correlated with contest intensity (Spearman's rho = 0.333, p = 0.002; Fig. S1).

## Discussion

Despite having both shorter hind femora and shorter pronota, wild-caught male *G. pennsylvanicus* had wider heads, greater maxillae spans, longer maxillae, and longer mandibles than females (Table 1) and were also more likely than females to have damaged mouthparts. This pattern of sexual dimorphism is consistent with the hypothesis that male heads, maxillae and mandibles evolved to be larger than females in response to male-male competition since male field crickets use their heads and mouthparts in escalated physical combat with other males [36], [41], [43], [44], whereas females do not [46]. It also confirms earlier observations of this species [48] and a recent study of the house cricket, *A. domesticus*, showing that males have larger heads than females [47].

In two experiments where we experimentally controlled for differences in male body size, we found that males with larger weaponry (i.e. heads, maxillae and mandibles) won more aggressive contests than males with smaller weaponry, although this was statistically significant in only the second experiment. Additionally, in both experiments, as the magnitude of the difference between males in head and mouthpart size increased, so too did the probability that the male with the larger head and mouthparts would win the agonistic encounter (Fig. 3). This pattern was even stronger when we considered only contests that escalated to grappling, the most intense level of male-male aggression (Fig. 4b). However, we failed to detect an effect of head and mouthparts on outcome in contests where males did not use these structures as weapons (Fig. 4a). In addition, we found no evidence that weapon size correlated with contest duration or intensity (Table S4). Therefore, our results suggest that male heads and mouthparts are not primarily signals used to assess RHP, but are weapons under selection for their use during direct physical combat.

Weapon size is positively correlated with weapon performance (e.g. bite force [66], [67]), which in turn is related to fighting success [66], [68]. In *G. pennsylvanicus*, the size of male weaponry is also likely correlated with weapon performance. For example, head width in crickets is probably related to bite force since the head capsule is largely filled with adductor muscles (KA Judge, pers. obs.) and a linear increase in head width would result in an exponential (squared) increase in muscle volume and therefore increased bite force. Indeed, this is the case in male tree weta, *Hemideina* spp., which have larger heads, greater adductor muscle volume and exert greater bite force than females [69]. Similarly, wider heads, longer maxillae, longer mandibles, and greater maxillae spans would lead to greater maximum gape, allowing males with larger weaponry to grasp the heads of opponents during combat. Males with large weaponry may also be able to impose more damage on rivals during escalated contests. The male bias in mouthpart damage found in wild-caught *G. pennsylvanicus* suggests that bite force may be sufficient to cause physical harm during contests (see also [1], [53]). The relationship between weaponry size, performance and the costs of escalated fights in field crickets could provide insights into the mechanisms by which animal contests are settled.

There is evidence that selection for larger weaponry through male-male combat found in *G. pennsylvanicus* may explain a broader pattern of morphology within North American field crickets. Recently, Jang et al. [70] studied aggression in males of four species of *Gryllus* field crickets: *G. fultoni*, *G. vernalis*, *G. pennsylvanicus* and *G. rubens*. They found that contests between males of both *G. pennsylvanicus* and *G. rubens* frequently escalated to grappling, whereas this level of aggression was never observed for *G. fultoni* or *G. vernalis* [70]. Interestingly, *G. fultoni* and *G. vernalis* have narrower heads than *G. pennsylvanicus* and *G. rubens* for a given body size (see Fig. 16 in [48]). These patterns suggest a central role for male-male competition in shaping morphology in a widely distributed and diverse group of animals.

An alternative, but not exclusive hypothesis for the pattern of sexual dimorphism seen in grylline field crickets is that head, maxilla and mandible size are under negative selection in females. This might arise if smaller mouthparts were more efficient at processing food since females are under strong pressure to maximize food intake because of the energetic demands of egg production, whereas males' energetic costs (e.g. singing, spermatophore production, fighting) are probably less than females'. Additionally, sex-specific optima for shared traits have the potential to result in intralocus sexual conflict [71]. There is evidence of sexual conflict over nutrient intake in field crickets. In *Teleogryllus commodus*, male and female reproductive performance peak at diets that differ in nutrient composition: high-carbohydrate, low-protein for males and an equal ratio of carbohydrate to protein for females [72]. Interestingly, when given a choice between diets that differed in the carbohydrate to protein ratio, males and females chose a similar diet that was intermediate between their fitness optima [72]. If *T. commodus* is sexually dimorphic in maxillae and mandibles, then this may provide a resolution to the conflict over nutrient intake if the mouthparts of each sex are more efficient at extracting the sex-specific optimum nutrient composition from the foods that they normally consume in the wild (e.g. smaller maxillae and mandibles were more efficient at extracting protein than larger maxillae and mandibles).

We did not detect an effect of weaponry size on contest duration or intensity (although these two variables were positively correlated, Fig. S1), and so were unable to find support for any of the models of contest assessment, all of which predict that such relationships will exist [29], [30], [31], [32], [34], [33], [35]. This could be because RHP due to weaponry size may not be assessed until males engage in direct physical combat. However, weaponry size was also not related to the duration of grappling in the most escalated fights (Table S4). Alternatively our measure of contest duration may not have reflected duration from the participants' perspective. For instance, in many of our contests before the encounter escalated, one or both males performed courtship song and backed toward their opponent in a posture that is more typical of male-female interactions. Winning and losing males were equally likely to display courtship behaviour (winners: 24/86, losers: 21/86, p = 0.729). It is not clear whether the courting individual recognized the sex of the other cricket in the arena, even when the opponent reacted aggressively. This may have resulted in measurement error in contest duration, particularly for some low intensity contests (see Fig. S1). However, because no one has studied the function of courtship during male-male interactions in field crickets, we do not feel justified in excluding portions of contests where courtship occurred as it may have a heretofore-unrecognized aggressive function.

The current results suggest that maxillae span is under positive selection through male-male competition in *G. pennsylvanicus*. Head width (and thus likely maxillae and mandible size) is also under positive linear selection via female choice (K. A. Judge, unpubl. ms). The apparent congruence between these two different mechanisms of sexual selection may be the reason that head width and mouthpart size are all positively allometric in males when compared with other morphological traits (Table S1, Fig. S2). In contrast, Bonduriansky and Rowe [73] showed that male head elongation in a piophilid fly, *Prochyliza xanthostoma*, is under conflicting sexual selection. Males with relatively elongated heads were at a disadvantage in their first fight with an opponent, although they were more attractive to females [73]. Head length (nor indeed any sexual trait) is not positively allometric in this fly [74]. The comparison of *G. pennsylvanicus* and *P. xanthostoma* highlights the need for studies of sexual traits within a group of related species, to draw conclusions about the different sexual selection pressures that shape morphology. Given their morphological diversity [48], diverse life histories [48], [62], [75], [76], alternative mating strategies [77] and the existence of a phylogeny [78], North American gryllines represent an ideal taxon to test comparative hypotheses concerning the evolution of allometries.

Finally, our results are consistent with reports of Chinese cricket fighting, which pointed to relatively larger heads (and thus larger maxillae and mandibles) as a trait that influences fight outcome [52], [53], [54]. Our experimental control of body size resembles Chinese cricket fights, where only contestants that are closely matched for body weight are pitted against each other [53], [54], [55]. Thus it is perhaps not surprising that we found a significant effect of weapon size on contest outcome. In contrast, Briffa [44] did not find an effect of mandible length on fight outcome in *A. domesticus*, although body size was a significant predictor of fighting success. Thus the influence of weapon size on contest outcome is probably weaker than that of overall body size. Another key difference between our study and that of Briffa's [44] is the relative rarity in *A. domesticus* of escalated contests in which males grappled – a much more common occurrence in *G. pennsylvanicus* (this study, K. A. Judge, pers. obs., see also [70]). Practitioners of Chinese cricket fighting use devices known as “ticklers” [53], [54] to lash the antennae of their cricket during a fight and thereby incite him to higher levels of aggression. As a result, most traditional Chinese cricket fights probably escalate to grappling – precisely the stage where our results suggest large weaponry would be most advantageous.

The long history of Chinese cricket fighting [52], [53], [54], [55], [56], [57], [58] coupled with the strong incentives to careful observation provided by gambling [53], [57], [58] have apparently resulted in a very detailed knowledge of cricket behaviour and morphology. Our results provide scientific validation of this ancient cultural knowledge. Interestingly, there are reports that the Chinese cricket fighting community in Philadelphia, USA uses *G. pennsylvanicus* to practice their sport [79]. Chinese cricket fighting has provided testable hypotheses concerning male weaponry (this study) and the neurobiology of fight experience [80], and may well yield further insights into animal behaviour [56].

## Supporting Information

Figure S1 Relationship between contest intensity (the maximum aggression level attained in each contest) and contest duration.
(0.25 MB TIF)

Figure S2 Matrix of scatterplots for the log transformed morphological variables showing sexual dimorphism in a sample of 151 males (grey Xs) and 75 females (black Os). Abbreviations are as follows: LgMxS = log maxillae span, LgMMxL = log mean maxilla length, LgMMdL = log mean mandible length, LgMdLA = log mandible length asymmetry, LgHW = log head width, LgPW = log pronotum width, LgPL = log pronotum length, and LgMFL = log mean femur length.
(1.60 MB TIF)

Table S1 Matrix of major axis (MA) slopes (above the diagonal) and Pearson correlation coefficients (below the diagonal) for the eight morphological variables (log transformed) measured on 151 male and 75 female wild-caught G. pennsylvanicus.
(0.04 MB DOC)

Table S2 P-values of tests for Experiment by Weaponry (PC1 and PC2) interaction effects on the dependent variables: contest outcome, contest duration and contest intensity.
(0.03 MB DOC)

Table S3 Number of contests in each experiment that attained a given intensity level.
(0.02 MB DOC)

Table S4 Spearman rank correlations between PC1 and PC2 scores (winning male, losing male, larger male and smaller male) and both contest duration and contest intensity for the pooled dataset (N = 86 contests) as well as for contests that did not escalate (N = 38) and those that escalated to grappling (N = 48, only for contest duration).
(0.03 MB DOC)
