# Supplementary figures and images for: Male Weaponry in a Fighting Cricket
Source: PLoS One. 2008 Dec 24;3(12):e3980. doi: 10.1371/journal.pone.0003980 (PMC2601036; doi:10.1371/journal.pone.0003980)

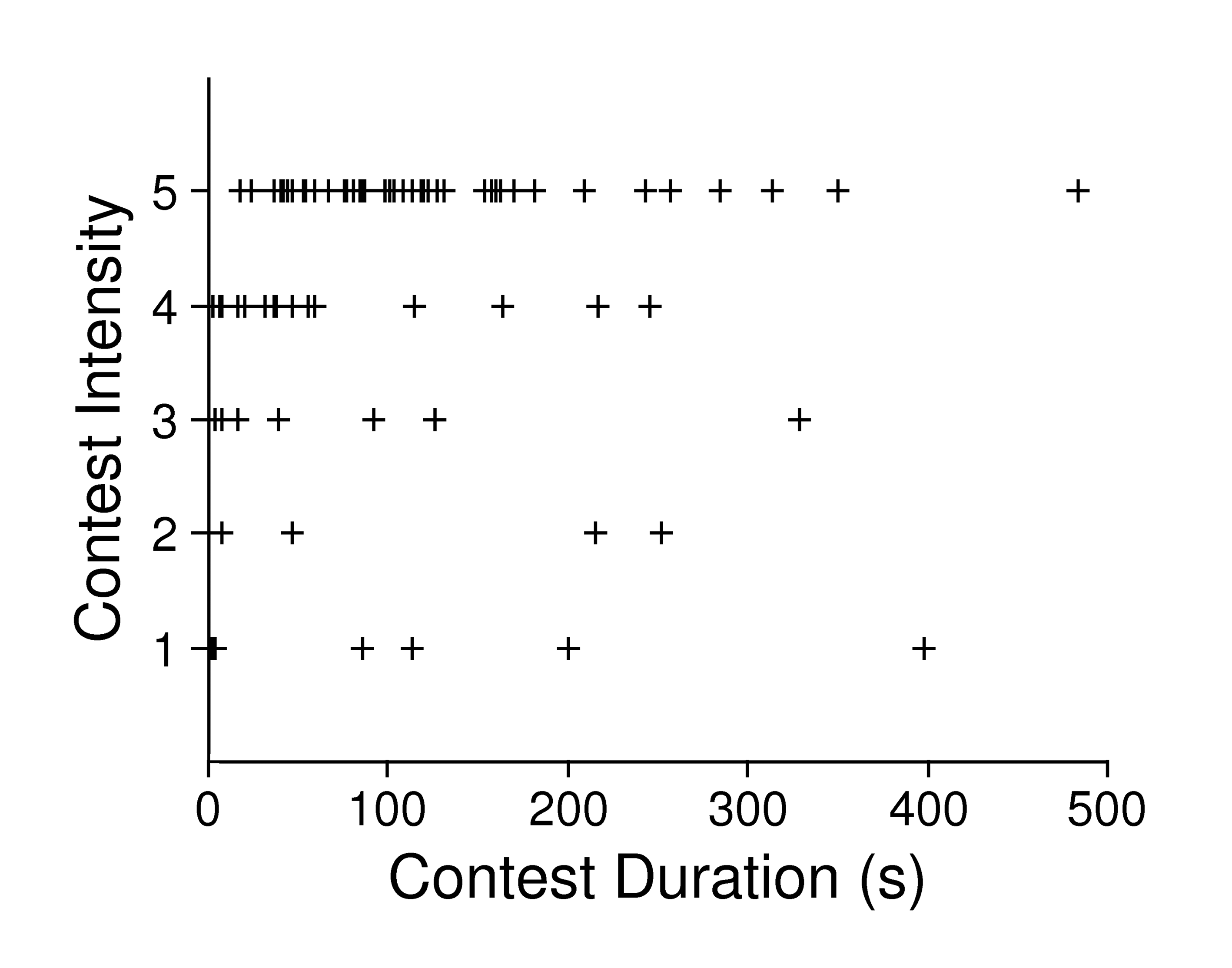

Supplement: Figure S1 — Relationship between contest intensity (the maximum aggression level attained in each contest) and contest duration. (0.25 MB TIF) [file pone.0003980.s001.tif]

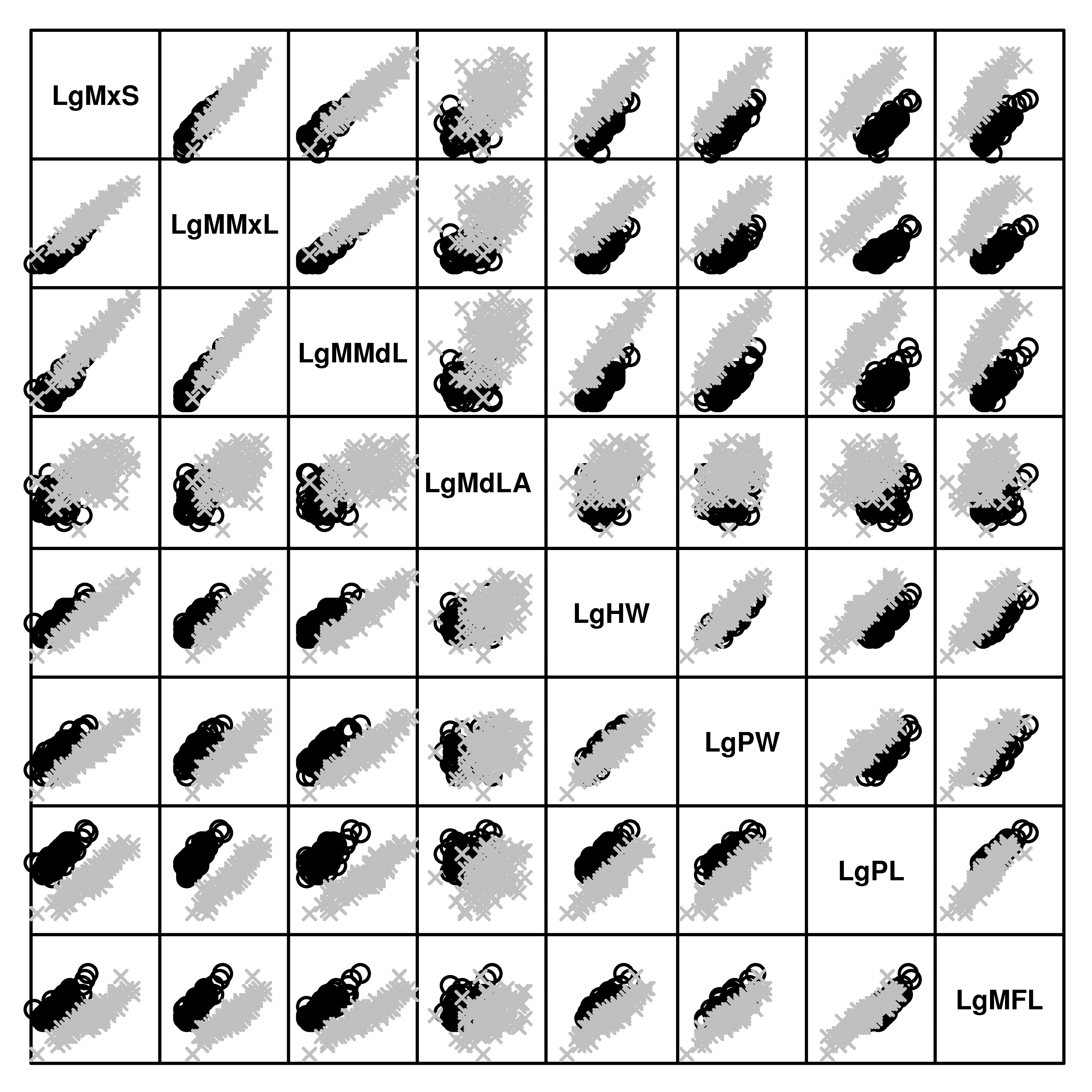

Supplement: Figure S2 — Matrix of scatterplots for the log transformed morphological variables showing sexual dimorphism in a sample of 151 males (grey Xs) and 75 females (black Os). Abbreviations are as follows: LgMxS = log maxillae span, LgMMxL = log mean maxilla length, LgMMdL = log mean mandible length, LgMdLA = log mandible length asymmetry, LgHW = log head width, LgPW = log pronotum width, LgPL = log pronotum length, and LgMFL = log mean femur length. (1.60 MB TIF) [file pone.0003980.s002.tif]
