# Supplementary material for: Male Weaponry in a Fighting Cricket
Source: PLoS One. 2008 Dec 24;3(12):e3980. doi: 10.1371/journal.pone.0003980 (PMC2601036; doi:10.1371/journal.pone.0003980)
Supplement: Table S1 — Matrix of major axis (MA) slopes (above the diagonal) and Pearson correlation coefficients (below the diagonal) for the eight morphological variables (log transformed) measured on 151 male and 75 female wild-caught G. pennsylvanicus. (0.04 MB DOC) [file pone.0003980.s003.doc]

**Table S1.** Matrix of major axis (MA) slopes (above the diagonal) and Pearson correlation coefficients (below the diagonal) for the eight morphological variables (log transformed) measured on 151 male and 75 female wild-caught *G. pennsylvanicus*.

| Variable | Sex | LgMxS | LgMMxL | LgMMdL | LgMdLA | LgHW | LgPW | LgPL | LgMFL |
| --- | --- | --- | --- | --- | --- | --- | --- | --- | --- |
| Log Maxillae Span | Males |  | 0.93(0.89-0.96) | 0.90(0.86-0.95) | 7.73(5.33-13.87) | 1.13(1.08-1.18) | 1.20(1.13-1.27) | 1.23(1.14-1.33) | 1.49(1.37-1.62) |
|  | Females |  | 1.01(0.92-1.11) | 0.98(0.83-1.14) | 8.75(3.99- -55.23) | 1.18(1.05-1.32) | 1.01(0.89-1.16) | 1.07(0.91-1.26) | 1.08(0.93-1.26) |
| Log Mean Maxilla Length | Males | 0.971 |  | 0.98(0.94-1.01) | 8.85(6.00-16.62) | 1.22(1.16-1.28) | 1.30(1.22-1.39) | 1.33(1.24-1.43) | 1.61(1.49-1.75) |
|  | Females | 0.928 |  | 0.97(0.88-1.06) | 10.38(4.23- -24.86) | 1.16(1.04-1.30) | 1.00(0.87-1.16) | 1.05(0.90-1.23) | 1.06(0.94-1.20) |
| Log Mean Mandible Length | Males | 0.958 | 0.977 |  | 10.43(6.73-22.96) | 1.25(1.19-1.31) | 1.34(1.25-1.43) | 1.37(1.27-1.47) | 1.65(1.53-1.79) |
|  | Females | 0.834 | 0.930 |  | 34.96(6.12- -9.50) | 1.21(1.06-1.37) | 1.04(0.88-1.24) | 1.11(0.91-1.35) | 1.11(0.96-1.28) |
| Log Mandible Length Asymmetry | Males | 0.343 | 0.327 | 0.283 |  | 0.15(0.09-0.22) | 0.15(0.08-0.22) | 0.16(0.09-0.24) | 0.19(0.10-0.29) |
|  | Females | 0.191* | 0.159* | 0.051* |  | 0.13(-0.04-0.31) | 0.09(-0.05-0.23) | 0.11(-0.04-0.26) | 0.09(-0.06-0.25) |
| Log Head Width | Males | 0.966 | 0.954 | 0.955 | 0.356 |  | 1.06(1.00-1.13) | 1.08(1.02-1.15) | 1.31(1.21-1.40) |
|  | Females | 0.894 | 0.898 | 0.882 | 0.173* |  | 0.86(0.75-0.98) | 0.90(0.79-1.03) | 0.92(0.81-1.04) |
| Log Pronotum Width | Males | 0.945 | 0.929 | 0.926 | 0.320 | 0.939 |  | 1.02(0.95-1.09) | 1.23(1.14-1.32) |
|  | Females | 0.874 | 0.850 | 0.803 | 0.148* | 0.869 |  | 1.06(0.87-1.28) | 1.07(0.89-1.29) |
| Log Pronotum Length | Males | 0.910 | 0.912 | 0.913 | 0.339 | 0.933 | 0.916 |  | 1.20(1.12-1.30) |
|  | Females | 0.827 | 0.835 | 0.763 | 0.168* | 0.865 | 0.775 |  | 1.01(0.88-1.16) |
| Log Mean Femur Length | Males | 0.893 | 0.898 | 0.899 | 0.308 | 0.912 | 0.909 | 0.912 |  |
|  | Females | 0.844 | 0.887 | 0.857 | 0.139* | 0.885 | 0.789 | 0.860 |  |

MA slopes and confidence intervals calculated using software for Model II regression provided by P. Legendre; http://www.bio.umontreal.ca/casgrain/en/labo/model-ii.html

95% confidence intervals are given in parentheses for the MA slopes.

p < 0.001 for all Pearson correlation coefficients except for those indicated by an asterisk (*) where p > 0.1
