# Supplementary material for: Male Weaponry in a Fighting Cricket
Source: PLoS One. 2008 Dec 24;3(12):e3980. doi: 10.1371/journal.pone.0003980 (PMC2601036; doi:10.1371/journal.pone.0003980)
Supplement: Table S2 — P-values of tests for Experiment by Weaponry (PC1 and PC2) interaction effects on the dependent variables: contest outcome, contest duration and contest intensity. (0.03 MB DOC) [file pone.0003980.s004.doc]

**Table S2.** P-values of tests for Experiment by Weaponry (PC1 and PC2) interaction effects on the dependent variables: contest outcome, contest duration and contest intensity.

| Dependent Variable | Weaponry | PC1 | PC2 |
| --- | --- | --- | --- |
| Contest Outcome | focal - rival | 0.886 | 0.151 |
| Contest Duration | Winner | 0.606 | 0.586 |
| Contest Duration | Loser | 0.640 | 0.210 |
| Contest Duration | Larger | 0.493 | 0.623 |
| Contest Duration | Smaller | 0.715 | 0.956 |
| Contest Intensity | Winner | 0.700 | 0.719 |
| Contest Intensity | Loser | 0.304 | 0.749 |
| Contest Intensity | Larger | 0.770 | 0.898 |
| Contest Intensity | Smaller | 0.913 | 0.413 |

We used the following statistics to test for Experiment by Weaponry interaction effects on: a) contest outcome – logistic regression (dependent variable [DV] = focal male won, continuous independent variable [IV] = difference in weaponry [focal – rival], categorical IV = experiment), b) contest duration – ANCOVA (DV = square-root of contest duration, factor = experiment, covariate = weaponry), and c) contest intensity – ANCOVA (DV = contest intensity, factor = experiment, covariate = weaponry).
