# Supplementary material for: Male Weaponry in a Fighting Cricket
Source: PLoS One. 2008 Dec 24;3(12):e3980. doi: 10.1371/journal.pone.0003980 (PMC2601036; doi:10.1371/journal.pone.0003980)
Supplement: Table S3 — Number of contests in each experiment that attained a given intensity level. (0.02 MB DOC) [file pone.0003980.s005.doc]

**Table S3.** Number of contests in each experiment that attained a given intensity level.

| Intensity Level | Code | Experiment 1 | Experiment 2 |
| --- | --- | --- | --- |
| No Apparent Aggression | 0 | 5 | 3 |
| Immediate Dominance | 1 | 6 | 4 |
| Antennal Fencing | 2 | 3 | 2 |
| Unilateral Maxillae/Mandible Spreading | 3 | 6 | 1 |
| Bilateral Maxillae/Mandible Spreading | 4 | 10 | 6 |
| Grappling | 5 | 22 | 26 |
| Total |  | 52 | 42 |
