# Supplementary material for: Male Weaponry in a Fighting Cricket
Source: PLoS One. 2008 Dec 24;3(12):e3980. doi: 10.1371/journal.pone.0003980 (PMC2601036; doi:10.1371/journal.pone.0003980)
Supplement: Table S4 — Spearman rank correlations between PC1 and PC2 scores (winning male, losing male, larger male and smaller male) and both contest duration and contest intensity for the pooled dataset (N = 86 contests) as well as for contests that did not escalate (N = 38) and those that escalated to grappling (N = 48, only for contest duration). (0.03 MB DOC) [file pone.0003980.s006.doc]

**Table S4**. Spearman rank correlations between PC1 and PC2 scores (winning male, losing male, larger male and smaller male) and both contest duration and contest intensity for the pooled dataset (N = 86 contests) as well as for contests that did not escalate (N = 38) and those that escalated to grappling (N = 48, only for contest duration).

| Variable | Contest Duration Pooled | Contest Duration Non-Escalated | Contest Duration Escalated | Contest Intensity Pooled | Contest Intensity Non-Escalated |
| --- | --- | --- | --- | --- | --- |
| Winner PC1 | -0.027  (10761) | -0.140  (395) | 0.002  (>20000) | 0.216^*^ | 0.136  (419) |
| Winner PC2 | 0.061  (2104) | 0.094  (883) | 0.149  (348) | -0.034  (6784) | 0.135  (425) |
| Loser PC1 | -0.038  (5430) | -0.096  (846) | -0.009  (>20000) | 0.126  (489) | 0.022  (16211) |
| Loser PC2 | -0.183  (229) | -0.146  (363) | -0.190  (212) | -0.098  (812) | -0.089  (985) |
| Larger PC1 | -0.063  (1972) | -0.100  (779) | -0.032  (7659) | 0.117  (568) | 0.054  (2686) |
| Larger PC2 | -0.111  (632) | -0.066  (1796) | -0.051  (3012) | -0.062  (2036) | 0.085  (1081) |
| Smaller PC1 | -0.001  (>20000) | -0.114  (599) | 0.029  (9327) | 0.221^*^ | 0.150  (343) |
| Smaller PC2 | -0.036  (6051) | -0.070  (1596) | 0.100  (779) | -0.099  (795) | -0.022  (16211) |

Values in parentheses are sample sizes required to reach statistical significance at the measured effect size and direction with power (1 – ß) = 80%.

^*^ p < 0.05 before sequential Bonferroni correction [82]
